# Time‐dependent effects of hypoxia on cell metabolism, signaling, and iron homeostasis in the hematopoietic progenitor model KG1a

**DOI:** 10.1002/2211-5463.13527

**Published:** 2023-06-01

**Authors:** Inès Nahoui‐Zarouri, Claire Léger, Ghina El Samra, Cécile Cottet‐Rousselle, Jean‐Marc Moulis

**Affiliations:** ^1^ Laboratory of Fundamental and Applied Bioenergetics (LBFA) and SFR Environmental and Systems Biology (BEeSy) University Grenoble Alpes and Inserm U1055 France; ^2^ Univ. Grenoble Alpes, CEA, IRIG France

**Keywords:** hypoxia, iron homeostasis, leukemia, myeloid progenitors, redox signaling

## Abstract

Lowered availability of oxygen in the micro‐environment of cells perturbs metabolic and signaling pathways. It affects proliferation, tissue morphology, and differentiation. Leukemia impairs maturation of hematopoietic progenitors: the immune system, healing, and erythropoiesis are weakened, thereby perturbing iron homeostasis and further lowering oxygen provision to tissues. Here, the time‐dependent molecular consequences of sudden hypoxia were studied in the KG1a model of immature hematopoietic progenitors. The oxygen tension of KG1a cells was abruptly lowered from the experimentally usual *ca*. 20 to 1%. Growth and key hubs of signaling, metabolism, and iron homeostasis were monitored by a combination of immunological methods and functional assays. The collapse of oxygen availability stopped proliferation after one generation. The number of cells then remained approximately constant over several days, including after anaerobic changes in the culture medium. Lowered oxygen resulted in transient increase of the hypoxia‐inducible factor 1α and of its REDD1 target, inhibition of mechanistic (or mammalian) target of rapamycin, decreased autophagy, altered cap‐dependent translation, and minimal repression of the already weak oxidative phosphorylation. These adjustments did not trigger important cellular iron fluxes since the cells relied on their internal iron stores to survive. In conclusion, the response of the KG1a cells to stringent hypoxia is varied, with some established hypoxia‐sensitive pathways exhibiting activation whereas others were unaffected. The results draw attention to the flexibility of the environmental adaptation of cancer cells. They suggest that thorough characterization of early leukemic blasts is warranted to propose informed treatments to patients.

## Graphical abstract

Hypoxia stops growth of KG1a cells, transiently increases the hypoxia‐inducible pathway, and inhibits oxidative phosphorylation, mTOR phosphorylation, and autophagy. Iron release from ferritin allows cells to survive for a while. These time‐dependent hypoxic adjustments of this immature hematopoietic cellular model emphasize the flexibility of cancer cells to changes in their micro‐environment and they provide leads to target their development.

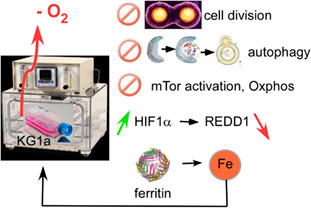

**Abbreviations**
AML: acute myeloid leukemia
ANOVA: Analysis of Variance
BSA: bovine serum albumin
HIF: hypoxia‐inducible factors
HMOX1: heme oxygenase 1
HMOX2: heme oxygenase 2
IRE: iron responsive elements
IRP: iron regulatory protein
PBS: phosphate buffered saline
PFKP: phosphofructokinase
qPCR: quantitative polymerase chain reaction
REMSA: RNA electrophoretic mobility shift assays
TFRC: transferrin transporter

The numerous links between hypoxia and iron homeostasis in mammalian cells and organisms have been increasingly appreciated over the last decades and regularly reviewed [1, 2]. These relationships and their deregulation are of importance in cancer initiation and development [3, 4].

The quantitatively major involvement of iron homeostasis in mammalian physiology is in erythropoiesis to generate red blood cells with their load of hemoglobin. In this process hypoxia plays a prominent role via the transcription factor subunit hypoxia‐inducible factor 2α that is modulated by iron regulatory protein 1 (IRP1) and that triggers erythropoietin synthesis [5, 6]. Expectedly, myeloid leukemia disturbs hematopoiesis and results in anemia.

However, the involvement of iron homeostasis in leukemia is not only associated with its role in erythropoiesis. On the one hand, iron availability is mandatory for cellular proliferation, and, on the other hand, deregulated iron handling may participate in deleterious reactions initiating carcinogenesis via the formation of iron‐catalyzed reactive oxygen species [7], the so‐called Fenton reaction. These species are very often claimed to be part of carcinogenesis by oxidizing various cellular components. But this cancer‐promoting mechanism may be counter‐balanced by the mechanism of programmed cell death called ferroptosis [8] that relies on the very same type of reaction. In ferroptosis, iron‐driven peroxidation of unsaturated lipids overflows the repair anti‐oxidant system of the oxidized lipids by glutathione peroxidase 4, which leads to death of damaged cells.

Of note, the interplay between iron homeostasis and hypoxia with production and resistance to reactive oxygen species [9] is of importance in leukemic stem cells with significant differences with normal hematopoietic stem cells in this respect [10].

It thus seems justified to further characterize the response of immature myeloid cells to hypoxia and the links with iron homeostasis. We here report studies with the KG1a model of such cells. The initially isolated KG1 cell line [11] has been widely used in the literature, and its characteristics recapitulate major features of minimally differentiated (CD34^+^) acute myeloid leukemia (AML) cells. KG1 cells maturate *in vitro* by treatment with cytokines, such as granulocyte‐macrophage CSF and TNF‐α, or phorbol esters [12]. However, the KG1 cell line includes cells at different stages of development, such as myeloblasts, myelocytes, and granulocytes [13]. To reduce this heterogeneity, a sub‐clone, called KG1a, was rapidly isolated from the parent line by agar‐plating KG1 cells and growing isolated colonies [13]. KG1a cells were immature and not responsive to growth factors and inducers of differentiation. Therefore, this KG1 subclone provides a stable hematopoietic primitive cell line that can be considered as an approximate cellular model of leukemic stem cells [14], including the CD34^+^CD38^−^ phenotype which is considered of poor prognosis in patients.

The aim of the study is to probe whether lowering oxygen availability in this cellular context may affect the use of iron as a trigger of proliferation, hence the ability of KG1a cells to multiply as well as with a very high oxygen tension of *ca*. 20% in air. Indeed, as an example of the relevance of such questioning, the clinically used drug deferasirox was developed as an iron chelator [15], although it may act in additional ways *in vivo* [16, 17]. Deferasirox was shown to enhance the level of reactive oxygen species and to efficiently kill leukemic CD34^+^CD38^−^ cells that display the phenotype of cancerous stem cells [18].

We show herein that not all metabolic and signaling responses of the KG1a cell line to a sudden shift to hypoxia obey previous observations with different cell systems under this environmental alteration. Growth of KG1a cells is rapidly stopped after about one generation, but without abrupt decline of the cell number over several medium changes. Some of the molecular details depart from a generally accepted view of the interdependence between important signaling and metabolic pathways such as those organized around the mechanistic (or mammalian) target of rapamycin (mTOR) hub or the glycolysis‐oxidative phosphorylation balance. Overall, our work illustrates the dynamic flexibility of leukemic cells to the modification of their microenvironment and the need to obtain the most detailed and timely description of patients' leukemic clones to succeed with specific informed treatments.

## Materials and methods

### Cell culture

The KG1a cell line was obtained from DSMZ (ACC 421; Leibniz Institute, DSMZ‐German Collection of Microorganisms and Cell Cultures GmbH, Braunschweig, Germany) and grown in RPMI 1640 (PAN Biotech, Aidenbach, Germany) containing 2 g·L^−1^ glucose with 10% (v:v) fetal bovine serum (Biowest, Nuaillé, France) at 37 °C in an incubator providing air (with approximately 20% O_2_) and 5% CO_2_. During propagation, cells were seeded at *ca*. 3 × 10^5^ cells·mL^−1^ and diluted or passaged at 10^6^ cells·mL^−1^. Hypoxia was implemented within the incubator in the Model 2 O_2_‐controlled *in vitro* cabinet (Coy Labs, Grass Lake, MI, USA) in which atmospheric O_2_ was displaced by nitrogen to dynamically adjust the O_2_ concentration that was continuously monitored. Although some preliminary experiments were carried out at 5% O_2_, the reported ones are at 1%. Where needed (see Results), cells were passaged in the absence of O_2_ by aseptically degassing the media and ensuring all anaerobic transfers with gastight needles and syringes under a nitrogen line [19]. Cell numbers were determined with the Logos Biosystems (Anyang City, Gyeonggi‐Do, Korea) Luna automatic cell counter after staining with trypan blue.

### Glucose and lactate assays

Glucose and lactate in the growth medium were measured with the Promega Glucose‐Glo (Cat. No J6021; Promega France, Charbonnières‐les‐Bains, Frnace) and Lactate‐Glo (Cat. No J5021) assays, respectively, according to the manufacturer's recommendations. The samples were diluted to fall in the assay detection range, and the metabolites' concentrations were calculated from reference curves after measuring luminescence in triplicate in white 384‐well plates with a multi‐mode reader (CLARIOstar; BMG Labtech, Ortenberg, Germany) using the preset UltraGlo filter.

### Cell cycle and immunofluorescence analysis

Cell cycle analysis was performed on a LSR Fortessa cell analyzer (Becton Dickinson, Le Pont de Claix Cedex, France) as previously detailed [20]: the procedure involved fixation in cold ethanol, RNA degradation with RNAse A, and labeling the DNA of the cells with propidium iodide. The results were analyzed with the modfit lt v3.2 software (Verity Software House, Topsham, ME, USA).

Immunofluorescence measurements were carried out with the relevant antibodies listed under Table 1. One million cells were collected by centrifugation and suspended in 0.5–1 mL of phosphate buffered saline (PBS). Formaldehyde was added to obtain a final concentration of 4% and fixation was carried out for 15 min at room temperature. Cells were recovered by centrifugation, washed with PBS, and suspended in 0.5–1 mL PBS. Nine volumes of ice‐cold methanol were added slowly with gentle vortexing to permeabilize cells, and the suspension was left on ice for 30 min. Cells were carefully washed several times with PBS to completely remove methanol and they were suspended at 2 × 10^6^ cells·mL^−1^ in PBS containing 5 mg·mL^−1^ bovine serum albumin (BSA). Tubes of 100 μL aliquots were prepared. In two of them, the primary antibody of interest (Table 1) was added at the 1 : 200 dilution, and the tubes were left 1 h at room temperature. Cells were recovered by centrifugation and washed twice with PBS. One tube, as well as another one with 4 × 10^5^ cells that were not exposed to the primary antibody, were treated with 100 μL of diluted DyLight 488‐conjugated Goat anti‐Rabbit IgG (H + L) secondary antibody (Thermo Fisher Scientific SAS, Illkirch Cedex, France, # 35552) prepared in BSA‐PBS buffer at the recommended 1 : 100 dilution. The second tube having reacted with the primary antibody was left in the BSA‐PBS buffer. All tubes were incubated for 30 min at room temperature, then washed twice with BSA‐PBS buffer, and the cells were finally suspended in PBS for analysis on the LSR Fortessa flow cytometer (Becton Dickinson). Each sample had thus 3 tubes, one containing cells having followed the complete staining procedure, and 2 others for negative controls, one without the primary antibody and the second without the secondary one. Measurements used the 488 nm sapphire laser and the corresponding fluorescence emission was selected with a 525/50 nm band‐pass filter.

**Table 1:** Antibodies used in the present work.

Antigen	Provider	Cat. No
Pan‐Actin	Cell Signaling Technologies	D18C11 #8456
β‐tubulin		9F3 #2128
Lamin B1		D9V6H #13435
p27 Kip1		D69C12 #3686
cyclin D1		92G2 #2978
HIF1α		D1S7W #36169
p‐S2448‐mTOR		D9C2 #5536
mTOR		7C10 #2983
p‐T37/46‐4E‐BP1		236B4 #2855
4E‐BP1		#9452
eIF4E		C46H6 #2067
PDK1 (as PDHK1 at CST)		C47H1 #3820
PFKP		D4B2 #8164
LC3A/B		D3U4C #12741
CD71 (Transferrin Receptor 1)		D7G9X #13113
FTH1		D1D4 #4393
IRP1	Home made	{[59] #518}
IRP2		{[36] #255}
HIF2α (EPAS1)	Novus Biologicals	NB100‐122
	Bethyl Laboratories	A700‐002‐Tor A700‐003
REDD1	Proteintech	10638‐1‐AP

### RT‐PCR and RT‐qPCR

KG1a cells were anaerobically harvested and the pellets were stored at −80 °C until use. RNA purification, reverse transcription, and qPCR amplification were performed as previously reported [21] with minimal changes. These changes were RNA purification with the RNeasy Plus Mini Kit (Qiagen, Germantown, MD, USA, Cat. No. 74134), and the qPCR reactions were implemented on the AriaMX instrument (Agilent Technologie France, Les Ulis, France). In each amplification for each sample, the cDNA of the RPLP0 (36B4, a ribosomal gene), of the hypoxanthine‐guanine phosphoribosyltransferase, and of the β‐actin genes were amplified as normalizers in parallel with the genes of interest. The normalizers were nearly stable under hypoxia for a given amount of cDNA template.

The oligonucleotides used to detect expression of the different genes are listed in Table 2.

**Table 2:** Oligonucleotides (5′–> 3′) used in qPCR experiments.

Gene	Forward and reverse primers
RPLP0 (ribosomal protein lateral stalk subunit P0)	GAAATCCTGGGTGTCCGCAATGTT
	AGACAAGGCCAGGACTCGTTTGTA
HPRT1 (HPRT, hypoxanthine phosphoribosyltransferase 1)	ATGGACAGGACTGAACGTCTTGCT
	TTGAGCACACAGAGGGCTACAATG
ACTB (β‐Actin)	GGATCAGCAAGCAGGAGTATG
	AGAAAGGGTGTAACGCAACTAA
RRM2 (ribonucleotide reductase regulatory subunit M2)	CACGGAGCCGAAAACTAAAGC
	TCTGCCTTCTTATACATCTGCCA
HIF1A (hypoxia‐inducible factor 1 subunit α)	ACGTGTTATCTGTCGCTTTGAGT
	TCGTCTGGCTGCTGTAATAATGT
HIF2A (EPAS1 – endothelial PAS domain protein 1)	CGTCCTGAGTGAGATTGAGAAG
	TCCTCCTTTAGCTTGGTGAATAG
HMOX1 (heme oxygenase 1)	CAGTGCCACCAAGTTCAAGC
	GTTGAGCAGGAACGCAGTCTT
HMOX2 (heme oxygenase 2)	GGAGCGCAACAAGGACCAT
	TCCTCCCAGTTTTCACCAAAGA
LDHA (lactate dehydrogenase A)	GCACCCAGTTTCCACCATGA
	TTCAAACGGGCCTCTTCCTC
SLC2A1 (solute carrier family 2 member 1; GLUT1: glucose transporter)	CTCCGGTATCGTCAACACGG
	GGCCACGATGCTCAGATAGG
TFRC (transferrin transporter; CD71; TFR1)	ACCATTGTCATATACCCGGTTCA
	GGCCTTTGTGTTATTGTCAGCAT

### Western blotting and RNA electrophoretic mobility shift assays

The procedures were previously described [21], except that western blots were processed with the BlotCycler‐automated western blot apparatus (Precision Biosystems, Mansfield, MA, USA) allowing the user to independently process six blots in parallel. Blots were developed with the Amersham ECL Prime Western Blotting Detection Reagent (GE Healthcare Life Sciences, Cat. No. RNP2232; https://one‐self.gehealthcare.fr/gehcstorefront/) and imaged on an ImageQuant LAS4000 imaging system (GE Healthcare Life Sciences). Guanidine solutions were used for successive probing of the same PVDF membrane [22] for soluble proteins. The lysates were normalized with the signal of actin, lamin B1, or tubulin depending on the probed size range and the expected intracellular localization of the protein of interest. The exception was the transferrin receptor protein, a membrane protein, that was detected on nitrocellulose membranes from the particulate fraction of the lysates and stained with Ponceau Red for normalization.

The antibodies are listed in Table 1.

Images of western blots were quantified with the ImageJ software (https://imagej.nih.gov/ij/). The background was generated with the “Subtract Background” function, by setting a value for the rolling ball radius which erased the bands and kept only the background. The generated background was saved and subtracted from the original image of the blot with the “Image Calculator” function.

The bands of the resulting images (8‐bits) were quantified following the procedure outlined in https://lukemiller.org/index.php/2010/11/analyzing‐gels‐and‐western‐blots‐with‐image‐j/, and the relative densities were calculated with reference to that of the “normoxic” sample, *that is* that of cells grown with 20% O_2_.

The RNA electrophoretic mobility shift assays (REMSA) used the human ferritin H 5′‐GGUUUCCUGCUUCAA*CAGUGC*UUGGACGGAACCC‐3′ (apical loop italicized) Iron Responsive Element. The synthetic probe (400 pmol) was 3′ labeled with biotinylated cytidine bisphosphate (1 nmol) by 40 units of T4 RNA ligase (Thermo Fisher Scientific) overnight at 16 °C in Tris‐Cl 50 mm pH 7.8, MgCl_2_ 10 mm, 10 mm DTT, 1 mm ATP, 15% (v:v) poly(ethylene glycol) 8000. The RNA was extracted with the chloroform: Isoamyl alcohol (24 : 1) mixture and the aqueous phase were precipitated and rinsed in 70% (v:v) ethanol. The concentration of the labeled probe was estimated by both RiboGreen (Thermo Fisher Scientific) RNA staining and dot blot analysis with streptavidin‐HRP reaction with chemiluminescence detection as described below. Reaction of the labeled probe (6 nm) with the IRP contained in 10 μg of proteins in cell lysates was carried out for 20 min at 37 °C in Hepes 10 mm pH 7.7, MgSO_4_ 3 mm, KCl 40 mm, glycerol 2%, DTT 1 mm, with addition of 2% β‐mercaptoethanol (v:v) when needed. Five mg·mL^−1^ heparin and then loading buffer (1/10^th^ volume of glycerol 50% + bromophenol blue 0.05%) were added, and the mixture was separated on cooled non‐denaturating 8% polyacrylamide gels run in 0.25 × Tris‐borate‐EDTA (TBE) at 13 500 V·min. The separated proteins were transferred onto Hybond N+ (Amersham) membranes with the semi‐dry Trans‐Blot system (Bio‐Rad, Marnes‐la‐Coquette, France) in 0.5 × TBE for 10 min at 25 V. After transfer, the excess buffer was quickly absorbed by blotting paper, and nucleic acids were cross‐linked on the membrane with a UV lamp (254 nm, 8 W) held at *ca*. 1 cm from the surface for 5 min. The detection of the biotinylated probe was carried out with the chemiluminescent nucleic acid detection module from Thermo Fisher Scientific (part number 89880) by successive baths of blocking buffer, stabilized streptavidin‐HRP conjugate interaction in blocking buffer, washing, and equilibration. The membrane was immediately processed with the ECL luminol enhancer–peroxide mixture (Amersham), and the generated luminescence was recorded with an ImageQuant LAS 4000 CCD camera (GE Healthcare Life Sciences). The obtained images were quantified as above for western blots.

### Statistical analysis

Results are reported as means ± SD (standard deviation). Means of two groups were compared with the Welch's *t*‐test. One‐way ANOVA followed by Holm‐Sidak tests were used for the comparisons between several groups. A value of *P* < 0.05 was considered as a statistically significant threshold for differences.

## Results

The promotion of tumors is generally associated with decreased availability of substrates [23] including oxygen, in most parts of solid tumors and plausibly in the limited space of the bone marrow in the case of leukemia. This general phenomenon is largely due to restricted vascularization and cellular crowding. Provision and diffusion of oxygen on a per‐cell basis are thus limited in the already low oxygen tension of the bone marrow [24]. It has to be noticed that the oxygen tension in the bone marrow is not homogeneous [24] and that the level of hypoxia is an important parameter in cancer development and resistance to therapy [25]. It thus appears warranted to further scrutinize the molecular adaptation of human cancer cellular models to hypoxia. It is acknowledged that the word *hypoxia* in such circumstances may not be accepted by all, since some consider that the oxygen partial pressure used here (1%) is close to the physiological one in the bone marrow [26]. In the following and for simplicity, the word *hypoxia* will be associated with 1% O_2_ and *normoxia* with 20% O_2_, even though the latter is far above the physiological oxygen tension of most tissues.

### Growth of hypoxic cells

**Fig. 1:**
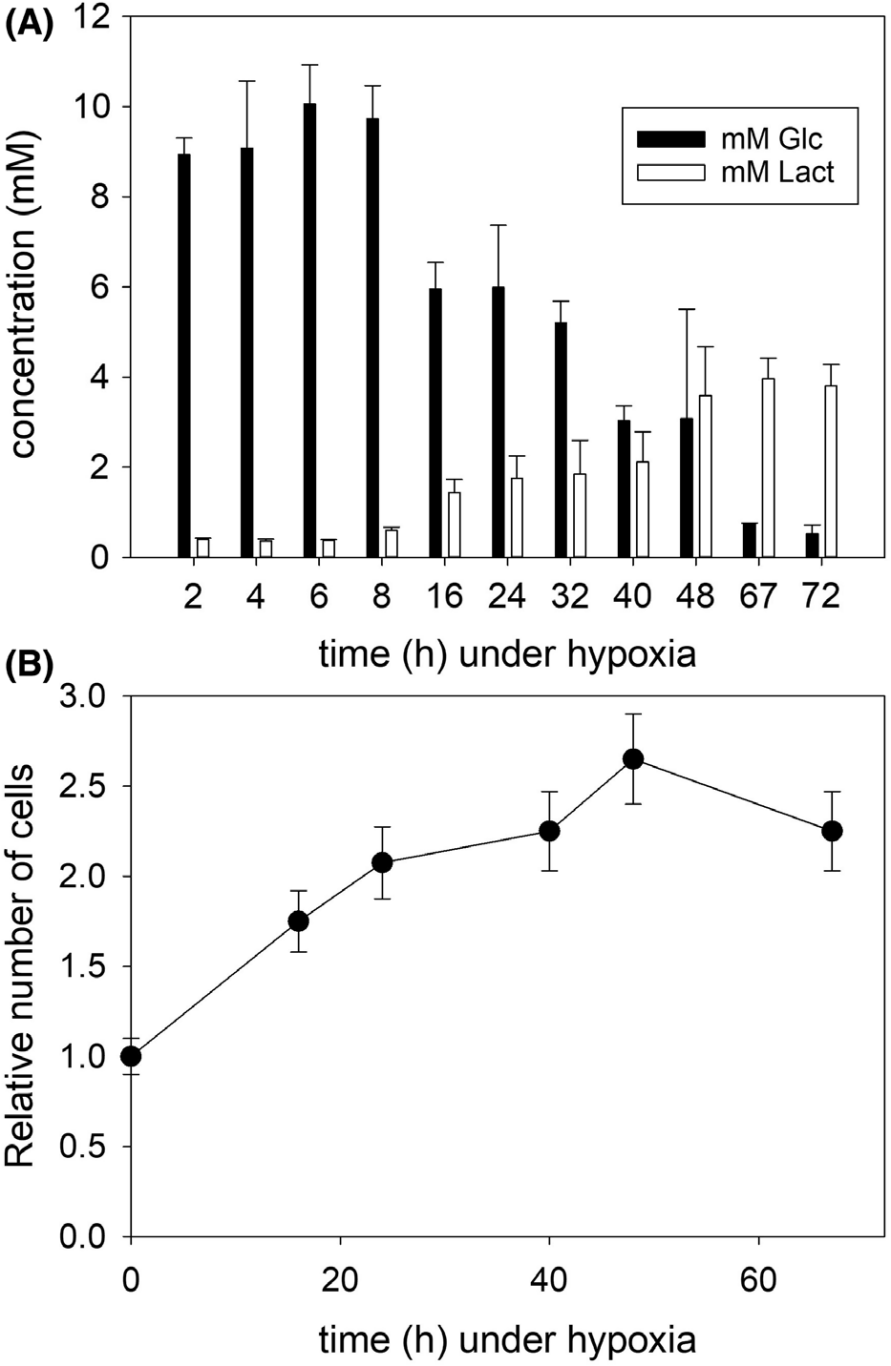
(A) Consumption of the carbon source (glucose) and production of the main metabolite (lactate) by KG1a cells under hypoxia. Concentrations of glucose (black bars) and lactate (white bars) were measured in the growth medium at 1% O_2_ as described in the experimental section. The plotted error bars correspond to the standard deviation of at least four measurements. (B) Evolution of the number of viable KG1a cells under hypoxia until glucose was used up. The error bars correspond to the standard deviation of at least three determinations of the number of viable and total cells in the culture medium.

The RPMI 1640 solution used to grow the KG1a cells contains 2 g·L^−1^ glucose, *that is* 11.1 mm. When cells were put under 1% O_2_, glucose gradually disappeared from the medium and was fully used up after *ca*. 3 days (Fig. 1A). In parallel, lactate increased (Fig. 1A). Meanwhile, the increase in cell numbers leveled off after *ca*. 30 h, *that is* approximately one generation (Fig. 1B). Such observations on concentrations of substrate and metabolite, as well as decreased proliferation, were expected from numerous previous studies carried out under hypoxia [27].

The following experiments aimed at quantifying the adaptation of the KG1a cells to their new hypoxic environment. Most of our experiments were carried out over a period of 3 days, unless otherwise stated, until the carbon source was exhausted and before it was replenished by a medium change. In those cases where cells were kept under hypoxia for longer than 3 days, the medium change was done under limited oxygen concentrations as mentioned in the experimental section.

**Fig. 2:**
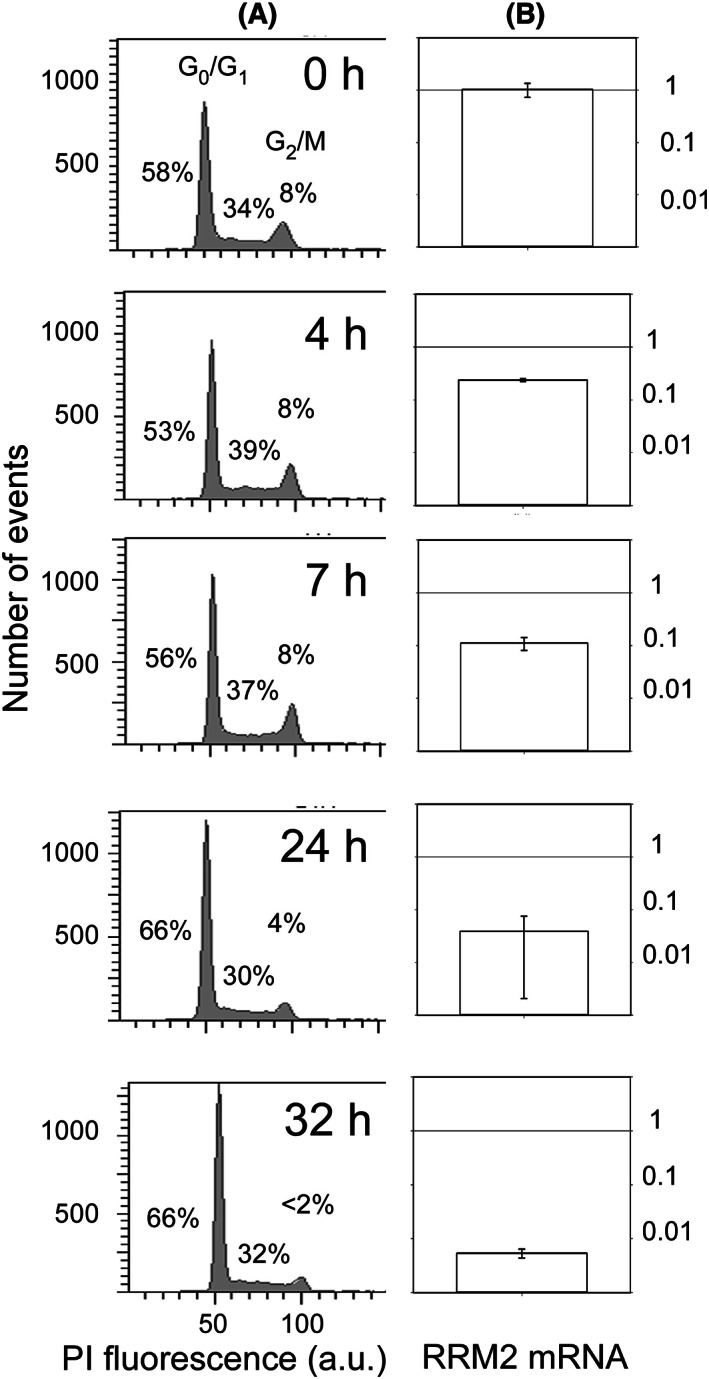
Changes in the cell population after shifting KG1a cells from 20 to 1% O_2_. (A) Representative example of the cell cycle populations. The cellular DNA was labeled with propidium iodide the fluorescence of which is plotted along the *x*‐axis and the number of cells along the *y*‐one. The indicated % shows the proportions of cells in the G1/S, S, and G2/M phases from left to right for each sample. (B) Amount, with SD, of RRM2 iron‐subunit mRNA relative to that under normoxia. Note the logarithmic scale used to draw the data.

Analysis of the cell cycle after shifting the cells from 20% O_2_ to 1% O_2_ indicated that the cycle was blocked at the G1/S restriction point. The G2/M population decreased, particularly after *ca*. 24 h, *that is* nearly one generation. DNA analysis of the flow cytometry histograms with the ModFit software indicated that the G2/M fraction fell from ca. 8% to *ca*. 1%, and the cellular population entering G0 or blocked at the G1/S checkpoint increased by approximately 10% in parallel, over the first 20–30 h (Fig. 2A). Ribonucleotide reductase is a key enzyme of the S‐phase by converting ribonucleotides (NDP/NTP) into the building blocks of DNA, namely deoxyribonucleotides (dNDP/dNTP). The catalytic subunit RRM1 depends on the iron‐containing subunit RRM2 that is upregulated in the S‐phase. In parallel to the G1/S blockade observed in the cell cycle, the level of ribonucleotide reductase (RRM2) mRNA decreased over time (Fig. 2B). The concentration of RRM2 mRNA reached levels that were several orders of magnitude smaller than that of strongly multiplying cells at 20% O_2_, but it did not completely disappear, even after anaerobically renewing the growth medium (not shown).

**Fig. 3:**
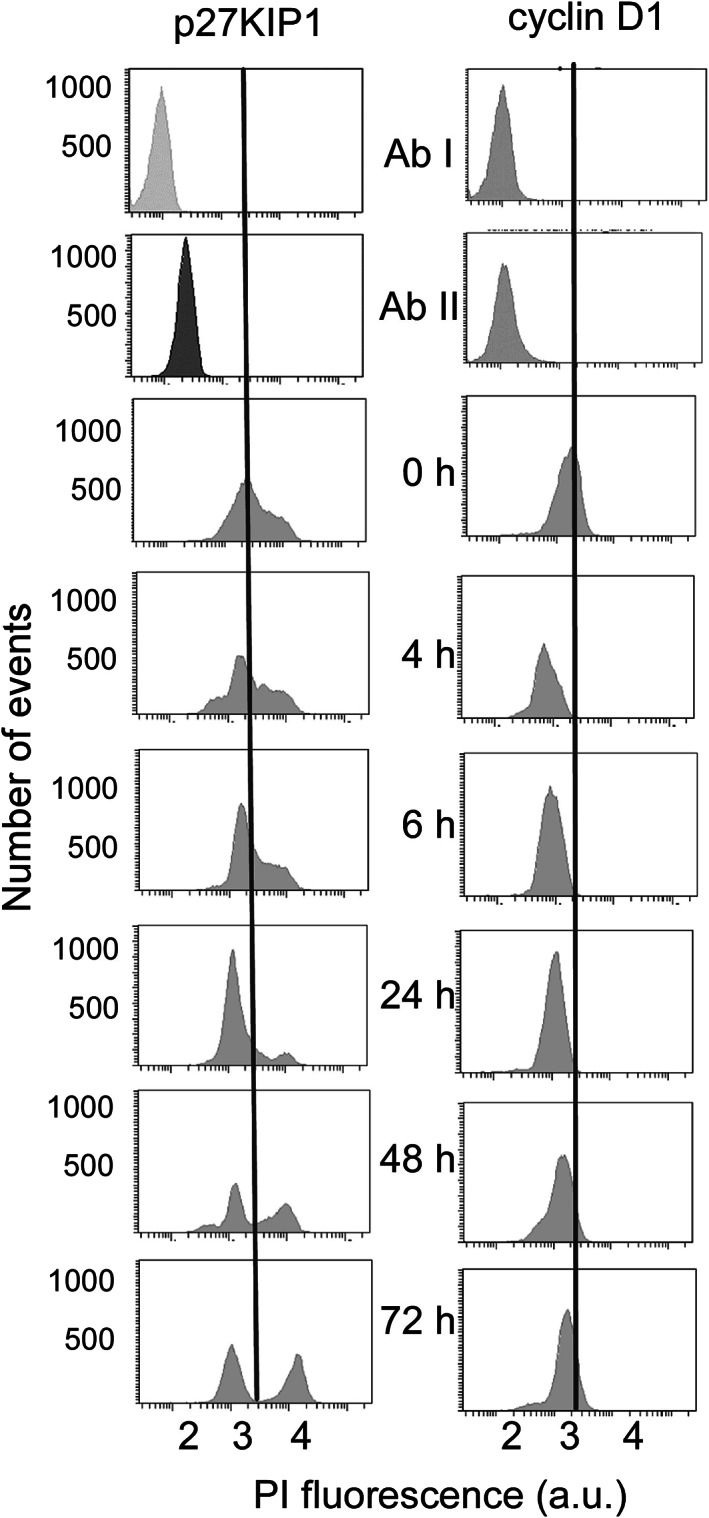
Representative immunofluorescence data for p27KIP1 (left) and cyclin D1 (right) after shifting KG1a cells from 20 to 1% O_2_. The fluorescence of the cells is plotted along the *x*‐axis with the tick labels indicating the powers of 10 (logarithmic scale). The top two panels show data obtained by omitting the secondary (goat anti‐rabbit IgG – DyLight 488‐conjugated) and primary (either p27KIP1 or cyclin D1) antibodies, respectively, in the labeling procedure to estimate the background fluorescence. The numbers in the middle of the figure indicate the time in hours spent by the cells under hypoxia.

To accumulate observations of the changes affecting the cell cycle after shifting from normoxia to 1% O_2_, the cellular amounts of the p27KIP1 (Cyclin‐dependent kinase inhibitor 1B) and the G1/S specific cyclin D1 proteins were measured by flow cytometry after labeling with antibodies. The best‐documented function of nucleus‐localized p27KIP1 is that of cyclin‐dependent kinase inhibitor that strongly contributes to G1 arrest of the cycle. Two populations of cells with different concentrations of the cycle inhibitor p27KIP1 were observed(Fig. 3). The one with the lowest concentration was already present in cells grown at 20% O_2_, and it decreased modestly, by a factor of <2 at most, under hypoxia. The identity of the species contributing to this peak is unknown. The second signal, that we assign to p27KIP1, corresponded to a larger concentration/fluorescence value, and it appeared as a shoulder of the previous one at 20% O_2_. It became clearly separated after 24 h, and the cell population containing it then increased, in fair agreement with the time dependence of the number of cells (Fig. 1B) and of DNA analysis of the cell cycle (Fig. 2A).

Cyclin D1, an interactor of p27KIP1, has also several roles, but the better‐known one is that of a regulator of the cyclin‐dependent kinase 4 (CDK) and CDK6 that trigger the transition from the G1 to the S‐phase of proliferating cells. In the case of cyclin D1, no major heterogeneity in the distribution of the label was found in immunofluorescence experiments. The basal signal at 20% O_2_ decreased under hypoxia by *ca*. 2.5‐fold after the shift in oxygen, with a tendency to recover toward the end of the incubation period of 3 days (Fig. 3). Later, after one hypoxic medium change, the same trend observed with hypoxia‐naïve cells was reproduced, with a decrease followed by partial recovery.

Overall, these data converge to indicate that the cell cycle is inhibited under hypoxia, but not altogether stopped, in agreement with the persistence of viable cells up to 10 days with regular, *that is* every 3 days, oxygen‐free medium changes. In the meantime, the proportion of cell debris increased. Consequently, when cells were anaerobically passaged, their total number was not decreased until *ca*. 5–6 days after being deprived of oxygen.

### Hypoxic molecular markers

**Fig. 4:**
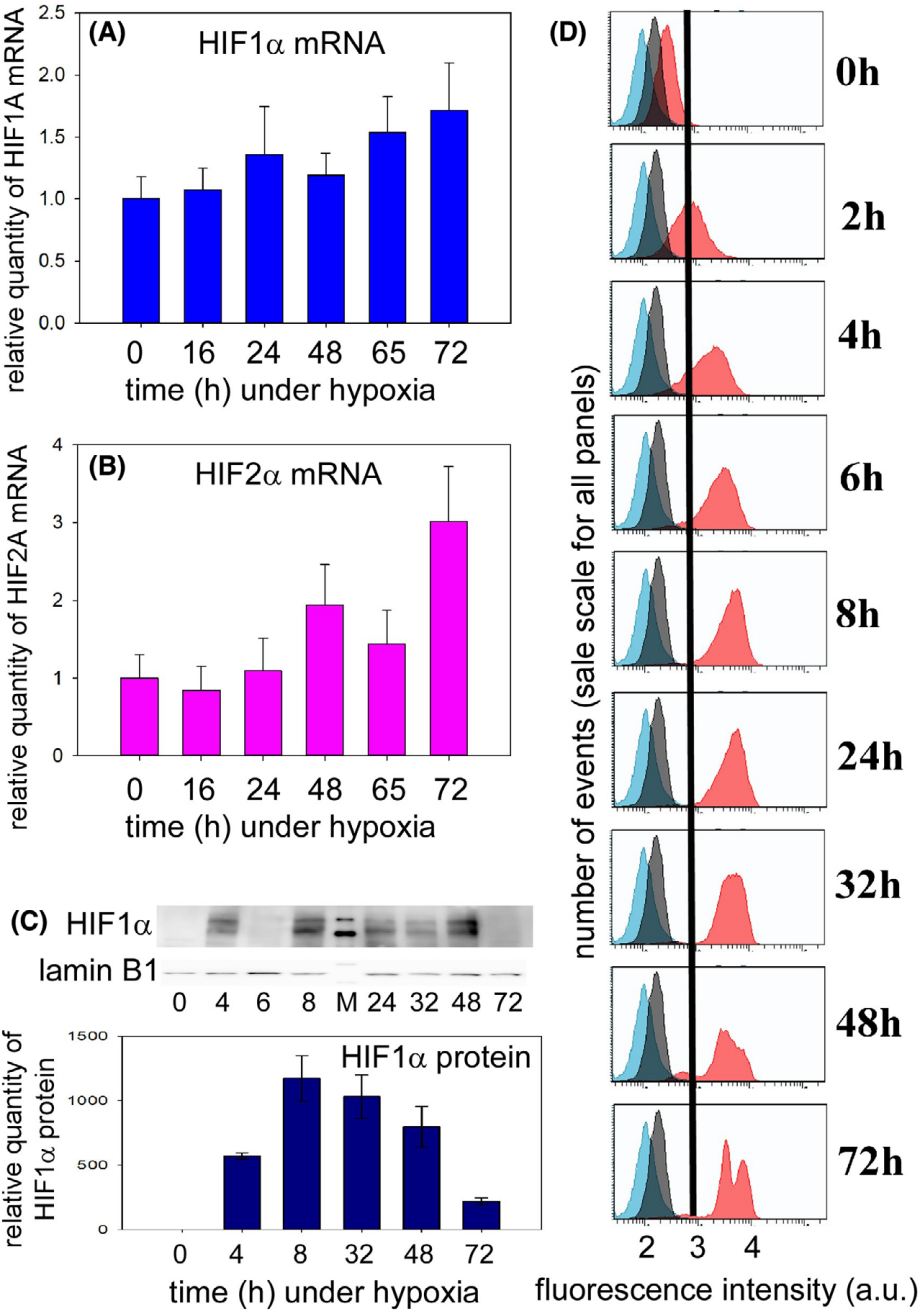
The hypoxia‐inducible factor subunit α in KG1a cells. (A) Changes of HIF1A mRNA concentration as a function of time after shifting from 20 (*t* = 0 h) to 1% O_2_. The reference value is that of the former condition used as calibrator and set to 1. (B) Same as A for HIF2A (EPAS1). (C) Representative western blot of HIF1α as a function of time under hypoxia and plot of the quantification of the HIF1α protein with SD for data at time points obtained from at least three different experiments. The molecular markers (lane M) in the upper, HIF1α, gel are at 100 and 120 kDa, and those in the lower, lamin B1, gel at 60 and 80 kDa. (D) Flow cytometry analysis of HIF1α after labeling with Dylight‐488 antibodies. The blue and gray traces as those obtained after omitting one of the antibodies as in Fig. 3. The red signal is obtained after completing the labeling procedure. The tick labels on the *x*‐axis are the powers of 10 of the fluorescence displayed by the cells.

The hypoxia‐inducible factors (HIF, mainly HIF1 and HIF2) are specific transcription factors that coordinate the cellular response to the lowering of oxygen, including in hematopoiesis [28]. The α subunits of these transcription factors (HIF1α and HIF2α or endothelial PAS domain protein 1, EPAS1) have a very short half‐life in the presence of oxygen as they then bind ubiquitin leading them to the proteasome. Indeed, oxygen is the substrate of the iron‐dependent enzymes proline hydroxylases with 2‐oxoglutarate as co‐substrate: O_2_ oxidizes specific proline residues into hydroxyl‐proline before ubiquitin conjugation. The HIF activity is mainly regulated at this post‐translational level, although modulation of the expression of these genes has also been described under some conditions [29, 30]. Therefore, the levels of HIF messengers were measured first. The mRNA of HIF1α and HIF2α was not found to vary much at any time after the shift from normoxia to hypoxia (Fig. 4A,B). One‐way ANOVA tests failed to return significant differences for any of the time points (*P* = 0.573 and 0.082 for HIF1α and HIF2α, respectively).

Second, at the protein level, western blots showed a rapid increase of HIF1α, from a hardly detectable band in normoxia, and a later decrease after a blunt maximum occurring between 8 and *ca*. 48 h, depending on the experiments (Fig. 4C). In addition, the fluorescence measured in flow cytometry experiments with labeled anti‐HIF1α antibodies confirmed the western blot results of a bell‐shaped plot as a function of time spent under hypoxia (Fig. 4D). In the same type of experiment, HIF2α was not reliably detected: in some cases, western blots with supposedly specific antibodies showed a band slightly above 100 kDa. However, we failed to get reliably significant variations of this band with the time spent by the cells in hypoxia, from 0 to 144 h, and we cannot be sure that the detected band corresponds to HIF2α. As a provisional conclusion, we state that HIF2α is not produced by KG1a cells under hypoxia. This is borne out by the qPCR data used to draw Fig. 4B that returned Cq values higher than 32 with cDNA templates giving values in the 26–27 range for HIF1α mRNA (Fig. 4A). This suggests that the so‐called oncogenic HIF2α axis does not play a major role in KG1a cells.

**Fig. 5:**
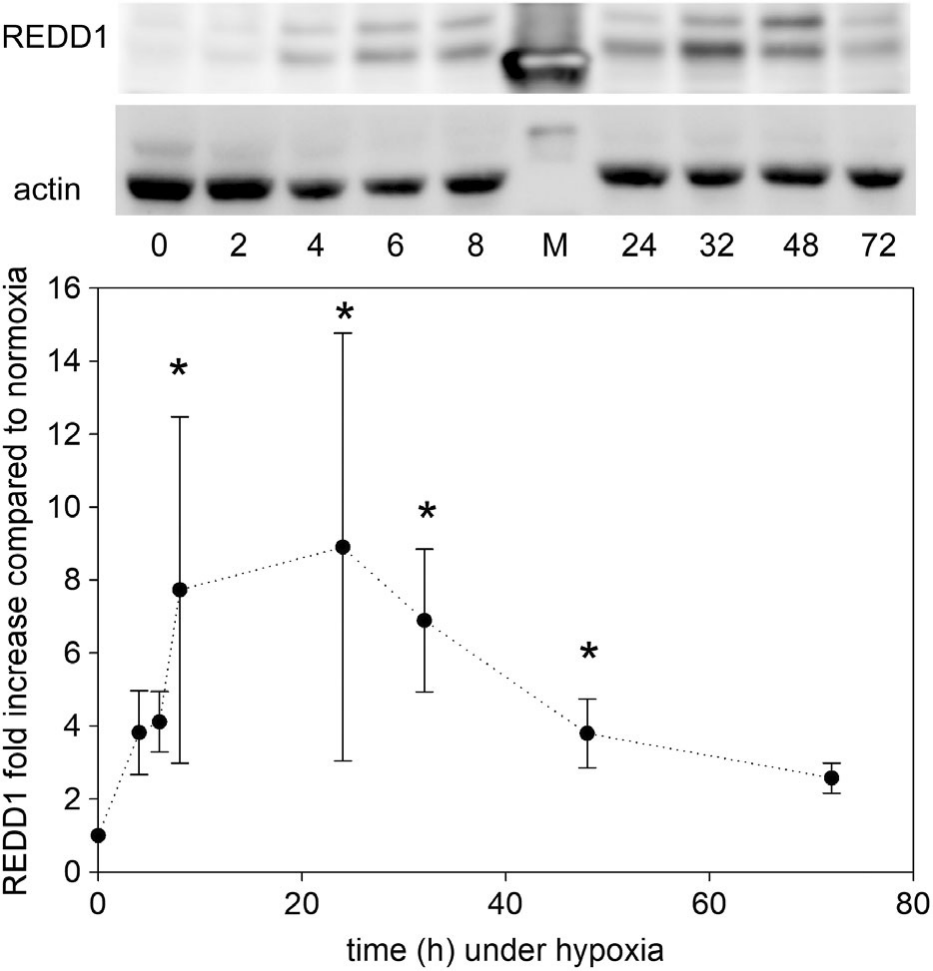
Evolution of the level of the REDD1 protein in KG1a cells under hypoxia. Top: Representative western blot of REDD1 as a function of time under hypoxia. The molecular marker (lane M) in the REDD1 gel is at 30 kDa, and that in the Actin gel at 60 kDa. Bottom: The intensity of the *ca*. 35 kDa protein detected with the anti‐REDD1 antibodies in western blots was integrated and plotted relative to the same band present at 20% O_2_ set to 1. The plotted error bars correspond to the standard error of at least three measurements. Kruskal‐Wallis one‐way analysis of variance on ranks, multiple comparisons versus *t* = 0 h with Dunn's method: * *P* < 0.015.

The HIF1 transcription factor has many targets, including REDD1, the product of the DDIT4 (DNA damage‐inducible transcript 4) gene. However, the REDD1 mRNA was not sensitive to hypoxia (not shown). The protein levels were evaluated by western blot (Fig. 5) and the band corresponding to REDD1 increased when cells were shifted to hypoxia (Fig. 5). One‐way ANOVA analysis indicated that the amount of REDD1 was significantly larger than under normoxia between 8 and 48 h after the shift to hypoxia (*P* < 0.012). After 2 days under hypoxia and until the next hypoxic medium change, the intensity of the REDD1 band slowly reversed back to the value found in normoxia. These data of mixed kinetics call for a combination of transcriptional and post‐transcriptional regulatory mechanisms acting on REDD1 under hypoxia [31].

### Signaling consequences of hypoxia

**Fig. 6:**
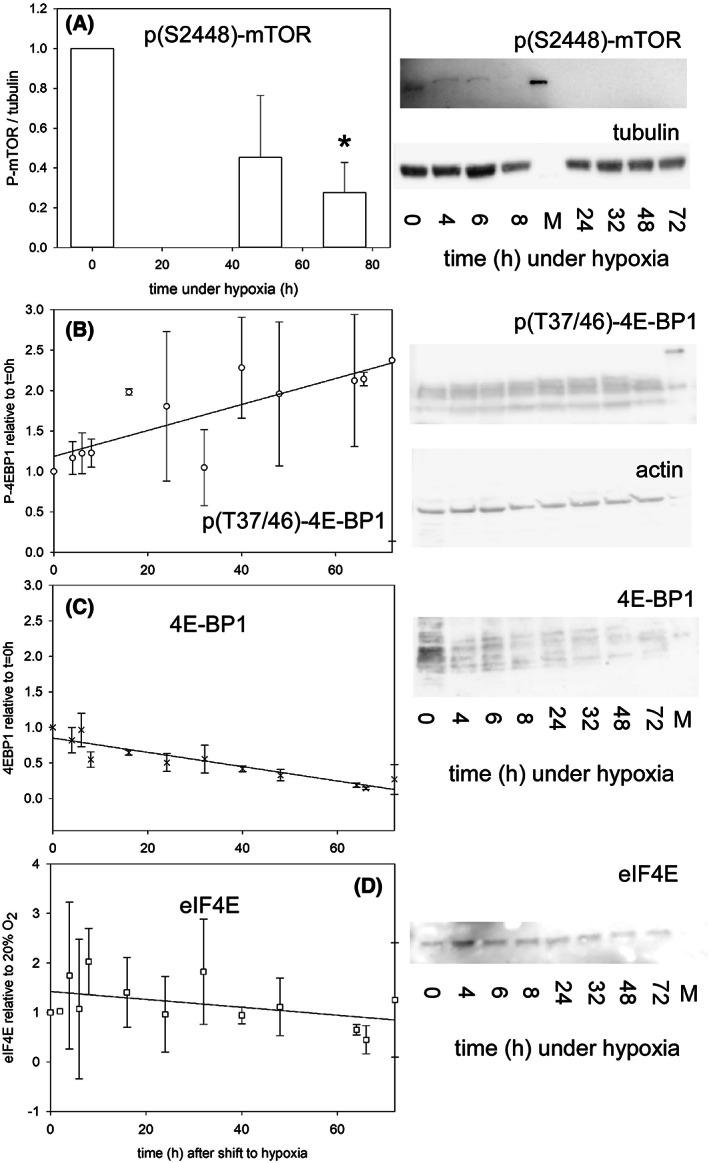
(A) Evolution of the phospho‐Ser‐2448 form of mTOR protein in KG1a cells under hypoxia. The intensity of the full‐length *ca*. 290 kDa protein detected in western blots was integrated and plotted relative to the same band present at *t* = 0, set to 1. The standard deviations of at least three measurements are plotted, and only time points with mean values different enough from the reference are drawn. *: Welch's *t*‐test, *P*‐value = 0.0141. An example of a western blot used to draw the plot is shown on the right. The band in the markers' lane (M) is at 220 kDa. (B) Evolution of the phosphorylated form of 4E‐BP1 in KG1a cells under hypoxia. At least 3 western blots were used to draw this panel with standard deviations plotted as well as the regression through the points with the following eq. 1.1865 + 0.016*t*, *r* ^2^ = 0.6475. (C) Same as B for the total 4E‐BP1 protein (regression: 0.8498–0.0101*t*, *r* ^2^ = 0.8430). (D) Same as B for the eIF4E protein. Panels B–D have the same time axis, and the relative amounts in B and C are also plotted on the same scale. Representative blots used to derive the plots in B–D and obtained from the same gel are shown on the right of the figure. The band in the markers' lane (M) is at 20 kDa.

REDD1 mediates inhibition of mTOR under hypoxia [32, 33]. Indeed, the phospho‐Ser‐2448 form of mTOR was found to decrease over time under hypoxia (Welch's *t*‐test between 0 and 3 days under hypoxia, two‐tailed *P*‐value = 0.0141; Fig. 6A), and the P(S2448)‐mTOR/mTOR ratio tended to decrease also (*t*‐test *P*‐value = 0.057). Of note, the implemented antibodies recognized two additional bands at *ca*. 85 and 55 kDa: these additional bands persisted beyond the disappearance of the full‐length P‐mTOR protein, but also with decreasing intensity over time. They likely correspond to degradation products of phosphorylated mTOR [34], which indicates turnover of phosphorylated mTOR under hypoxia. This implies that P(S2448)‐mTOR disappearance was largely due to proteolysis in addition to de‐phosphorylation.

The signaling hub mTOR regulates protein translation by integrating a variety of signals, such as nutrient availability or the presence of growth factors among others, in addition to hypoxia [35]. In the present conditions, the de‐repression of translation was assessed by measuring Thr 37/46 phosphorylation of the 4E‐BP1 inhibitor of the eIF4E initiation factor (Fig. 6B). Indeed, phosphorylation of 4E‐BP1 releases it from the eIF4E activator protein of the eIF4F pre‐initiation complex. Phosphorylated 4E‐BP1 was found to increase over time (regression: 1.1865 + 0.016*t*, *r* ^2^ = 0.6475, where *t* is the time). However, the total 4E‐BP1 protein (Fig. 6C) decreased consistently (regression: 0.8498–0.0101*t*, *r* ^2^ = 0.8430), with the consequence that the P‐4E‐BP1/4E‐BP1 ratio regularly increased under hypoxia. The evolution of (P‐)4E‐BP1 occurred with nearly the same amount, within a factor 2 at most, of eIF4E, the 4E‐BP1 interactor (Fig. 6D) (regression: 1.4246–0.008*t*, *r* ^2^ = 0.2117). Taken together, the measurements of these cap‐dependent components of the translation machinery suggest some increase of eIF4F complex‐dependent translation under the present hypoxic conditions.

### Stress markers

**Fig. 7:**
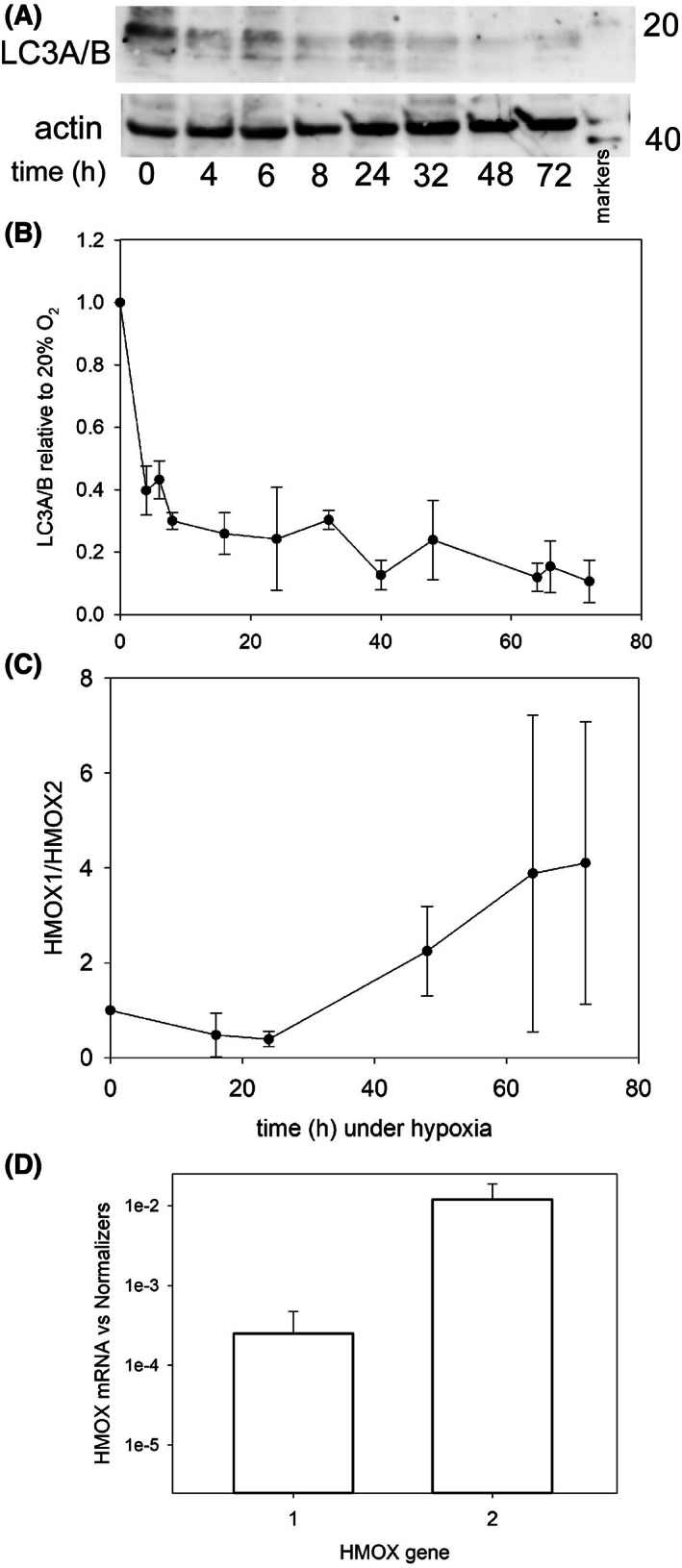
(A) Representative western of the LC3A/B bands present in KG1a cells. The time spent under hypoxia is indicated below the blots, with the position of the mass markers in kDa on the right of the panel. (B) Plot of the cumulative data obtained for LC3A/B. The intensity of the LC3A/B bands in different experiments was integrated and plotted relative to the signal displayed by cells at 20% O_2_ (*t* = 0 h) which was set to 1. The plotted error bars correspond to the standard error of at least three measurements. (C) Plot of the HMOX1/HMOX2 ratios under hypoxia. The levels of mRNA were measured by RT‐qPCR under hypoxia and calibrated to the value of cells grown at 20% O_2_ (*t* = 0 h). The HMOX1/HMOX2 ratio of the latter was arbitrarily set to 1. One‐way ANOVA analysis of all time points did not evidence statistical variations (*P* = 0.269). The plots in B and C have the same time scales. (D) Comparison of the expression of the HMOX1 and HMOX2 genes. At each time point under hypoxia, the levels of mRNA were measured by RT‐qPCR and normalized to the reference genes. Bars are for HMOX1 (left) and HMOX2 (right) from the pooled data. Note the log scale of the *y*‐axis.

The cellular stress induced by the shift from 20 to 1% O_2_ might be accompanied by autophagy that could explain at least part of the observed cellular degradation. To address this point, we used the microtubule‐associated proteins 1A and B (MAP1A/B; LC3A/B) as markers of autophagy since these proteins, lipidated LC3‐II in particular, are involved in the assembly of autophagosomes. Instead of the increase of at least one of the LC‐3 bands that would be the hallmark of enhanced autophagy, all bands of the LC‐3 isoforms rather decreased statistically (One‐way ANOVA *P* < 0.001) over time spent under hypoxia (Fig. 7A,B). It can thus be concluded that autophagocytosis is not responsible for KG1a growth stalling under hypoxia.

Keeping with the possible stress experienced by the cells deprived of oxygen, the transcriptional response mediated by heme oxygenase was evaluated. Neither the inducible gene heme oxygenase 1 (HMOX1) nor the constitutive one heme oxygenase 2 (HMOX2) showed significant changes of expression over time, and the HMOX1/HMOX2 ratio did not significantly (One‐way ANOVA *P* = 0.269) vary (Fig. 7C). Furthermore, the mRNA levels of HMOX1 remained far, *ca*. 50‐fold, and statistically (*t*‐test *P* < 0.001) lower than those of HMOX2 (Fig. 7D).

These data indicate that the cells did not experience a major stress, inducing HMOX1 that remained at hardly detectable levels, or enhanced autophagocytosis, by drastically lowering the oxygen tension.

### Metabolic markers

In many cases, the decrease of oxygen availability has been associated with a metabolic switch from energy production by oxidative phosphorylation to glycolysis. The predominance of glycolysis in many cancer cells has also been demonstrated, even in the presence of oxygen, hence receiving the designation of aerobic glycolysis. Molecular markers of the reactions generating cellular energy have been probed here for KG1a cells after shifting the oxygen tension from 20 to 1%.

**Fig. 8:**
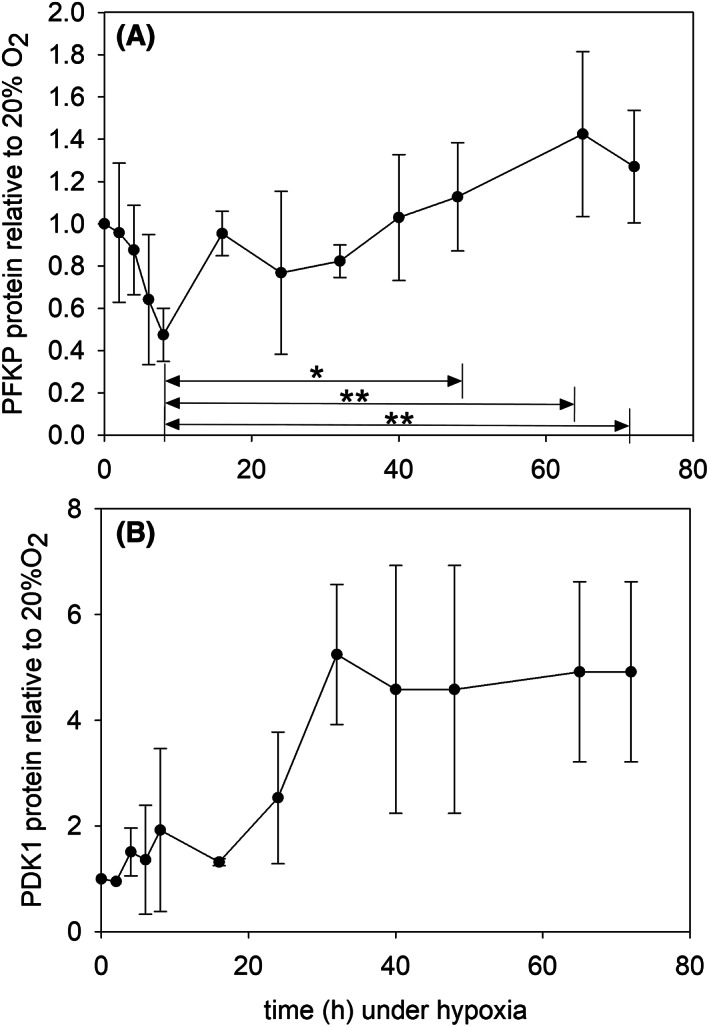
(A) Plot of the cumulative data obtained for PFKP. The intensity of the bands in western blots was integrated and plotted relative to the signal displayed by cells at 20% O_2_ which was set to 1. The plotted error bars correspond to the standard deviation of at least three measurements. One‐way ANOVA pairwise comparisons *P < 0.05; **P < 0.001. (B) Plot of the cumulative data obtained for pyruvate dehydrogenase kinase. The data obtained with PDK1 were treated as in A with PFKP. The differences in the mean values are significant with *P* = 0.004.

Phosphofructokinase (PFKP) phosphorylates fructose 6‐phosphate into fructose 1.6‐bisphosphate in the rate‐limiting step of glycolysis. In the present case, the level of PFKP decreased rapidly after switching from 20% O_2_ to hypoxia (Fig. 8A). However, this decrease was relatively modest, by an approximate 2‐fold factor, and it did not last long as the cellular content of PFKP slowly recovered its normoxic value over time. One‐way ANOVA picked significant differences (*P* < 0.001) between the lowest value, 8 h after shifting to hypoxia, and those above 60 h of hypoxia. These data thus indicate that glycolysis is quickly repressed by oxygen withdrawal. However, KG1a cells re‐implement glycolysis as a way to produce energy after a few hours under hypoxia at a level similar to that observed under abundant oxygen.

Fulling of the mitochondrial tricarboxylic acid (Krebs) cycle by pyruvate is inhibited by the specific kinase encoded by pyruvate dehydrogenase kinase (PDK1). This protein steadily increased after the hypoxic shift (Fig. 8B) with statistical significance (One‐way ANOVA *P* = 0.004).

In additional experiments, the lactate dehydrogenase mRNA was measured since the encoded enzyme diverts pyruvate from mitochondrial use by the tricarboxylic acid (TCA) cycle. This messenger did not significantly change under hypoxia. Also, the transcriptional regulation of the glucose importer SLC2A1 (GLUT1) depends on HIF1α. In the present conditions, it was weakly sensitive to hypoxia and it showed a modest increase of less than 2‐fold after 24 h at 1%O_2_ up to 3 days, but the increase did not reach statistical significance.

### Iron homeostasis under hypoxia

**Fig. 9:**
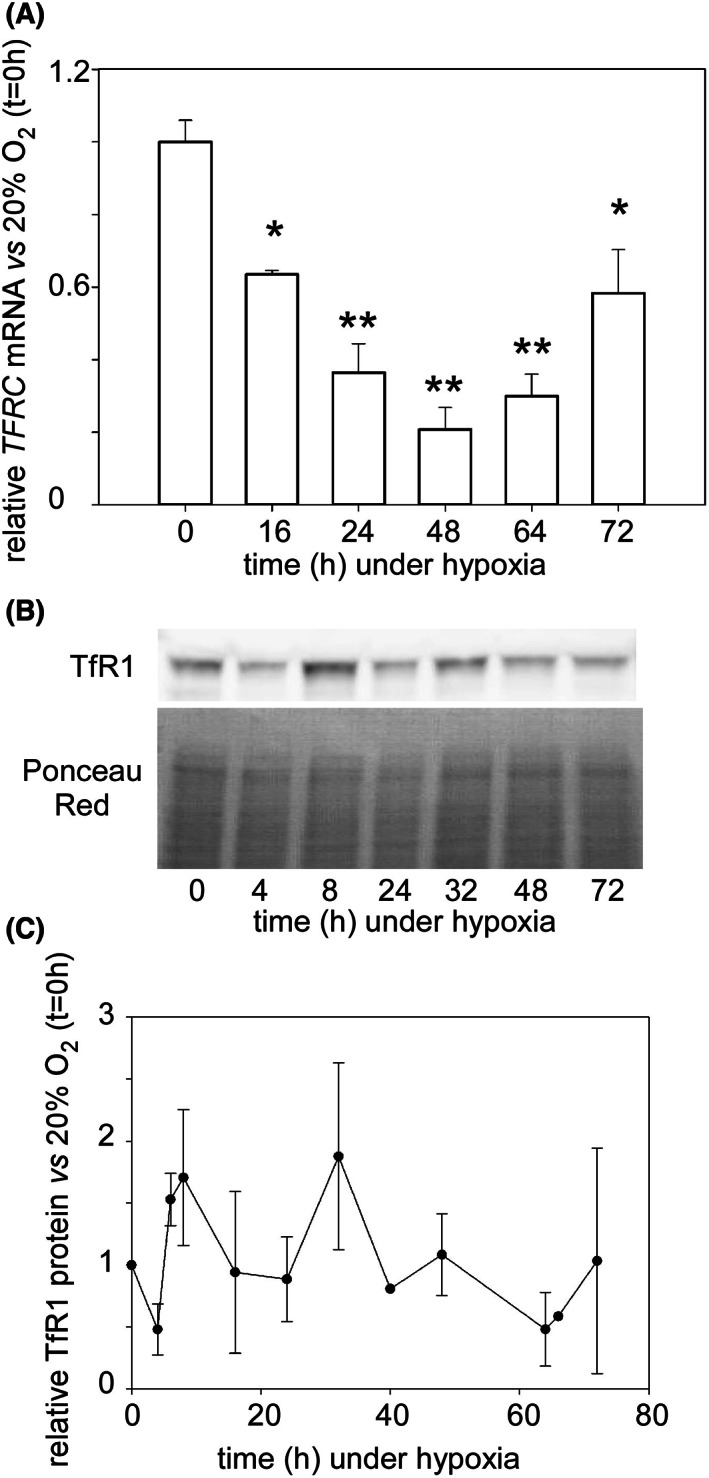
(A) Relative quantity of the TFRC transcript. The amount of transferrin receptor mRNA was measured by RT‐qPCR for all time points reported elsewhere in this study, as detailed in the experimental section. Cumulative data with standard deviations showing significant differences in the mean values in pairwise one‐way ANOVA comparisons with cells grown at 20% O_2_ (*P < 0.05 and **P < 0.001) are plotted. (B) Representative western blot of transferrin receptor 1 in KG1a cells. The transferrin receptor bands were calibrated with the intensity of the proteins in each lane of the membrane colored with Ponceau red. (C) Plot of the cumulative data obtained for transferrin receptor 1. The intensity of the bands in western blots was integrated and plotted relative to the signal displayed by cells at 20% O_2_ which was set to 1. The plotted error bars correspond to the standard deviation of at least three measurements.

When considering the growth of cells, and particularly cancerous ones, the availability of nutrients is of obvious importance. Among them, the essential metal iron is prominent and the changes occurring to regulatory elements of iron homeostasis upon applying hypoxia were monitored. Physiological cellular iron import is carried out by the transferrin receptor encoded by the transferrin transporter (TFRC) gene in most cells and tissues, including hematopoietic cells. The concentration of the transferrin receptor at the plasma membrane is regulated at several levels. Concerning the expression of the gene, down‐regulation was observed between application of hypoxia and *ca*. 60 h later, with partial recovery afterward (Fig. 9A). However, when the transferrin receptor protein was measured by western blots (Fig. 9B), no significant differences, as compared to the concentration under 20% O_2_, were found at any time after putting the cells under 1% O_2_, until glucose exhaustion after 3 days (Fig. 9C).

**Fig. 10:**
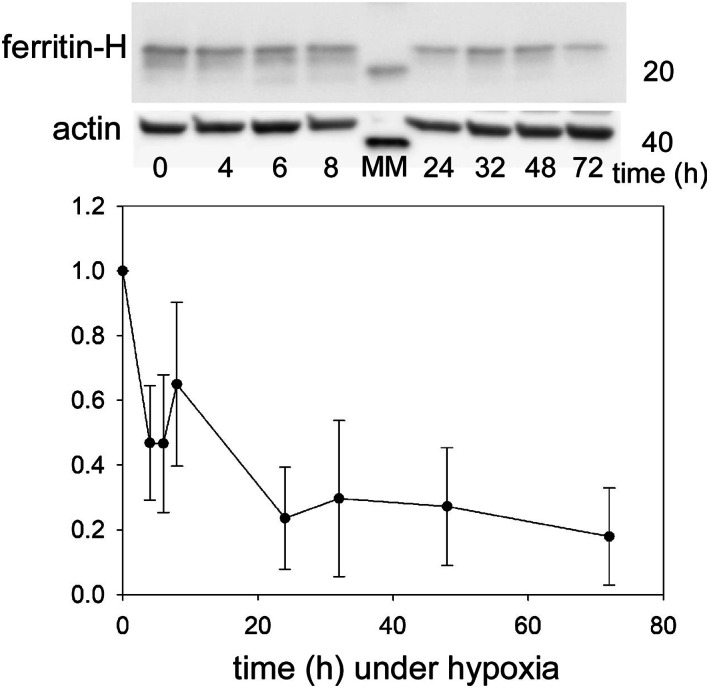
Top: Representative western blot of ferritin‐H in KG1a cells. The time spent under hypoxia is indicated below the blots. The central lane contains mass markers with their sizes in kDa on the right of the panel. Bottom: Plot of the cumulative data obtained for ferritin‐H. The intensity of the bands in blots such as the ones shown in ‘A’ was integrated and plotted relative to the signal displayed by cells at 20% O_2_ (*t* = 0 h) which was set to 1. The plotted error bars correspond to the standard deviation of at least three measurements. The differences in the median values are statistically significant (one‐way ANOVA *P* = 0.009).

Ferritin is the intracellular iron storage protein. The concentration of the H subunit containing the ferroxidase site of the protein which is involved in iron loading was found to decrease in experiments such as the ones used to draw Fig. 9. Such decrease reached statistical significance according to the one‐way ANOVA test (*P* = 0.009) after *ca*. 1 day under hypoxia (Fig. 10). Therefore, the needs for iron under hypoxia appear to be fulfilled by emptying the cellular stores from ferritin, rather than by *de novo* import through the transferrin receptor.

**Fig. 11:**
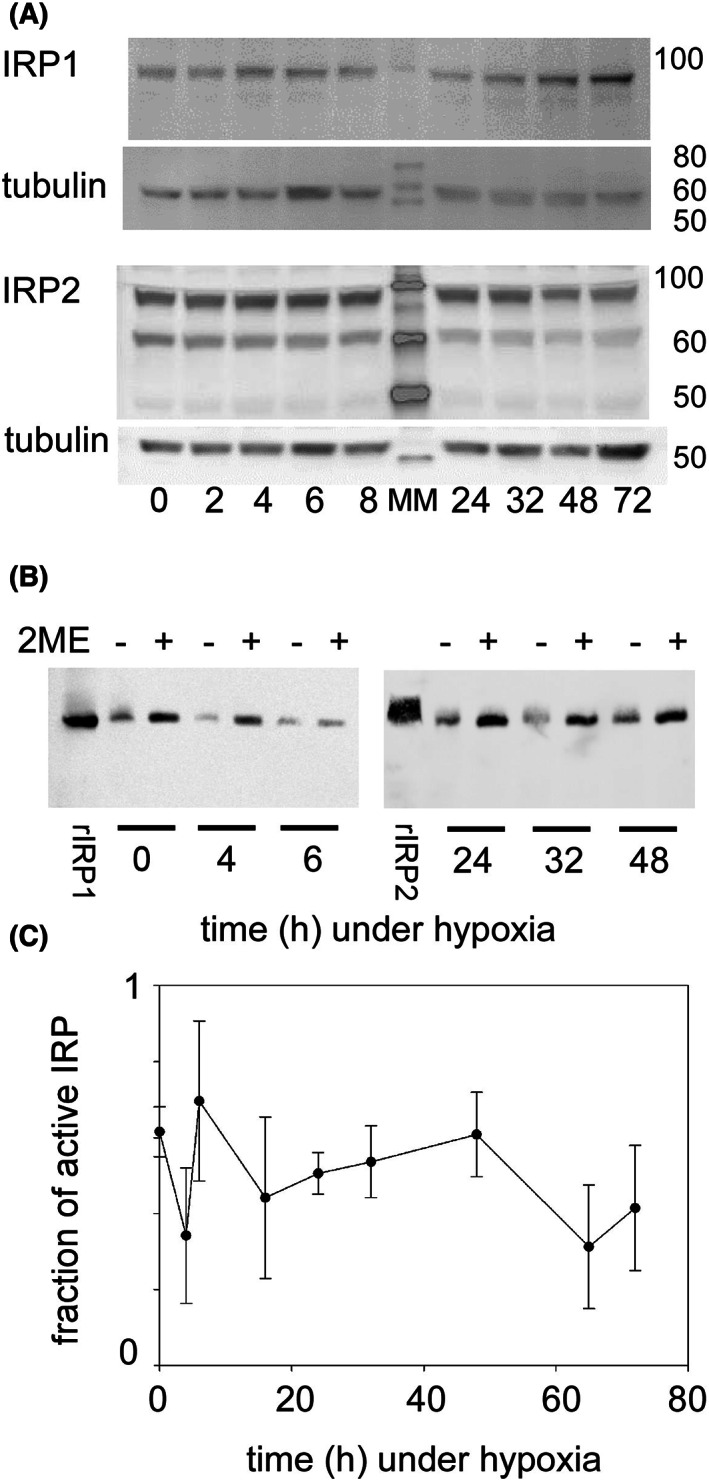
(A) Representative western blots of IRP1 and IRP2 in KG1a cells. The time spent under hypoxia is indicated below the blots. The central lane contains mass markers with their sizes in kDa on the right of the panel. (B) Example of a RNA electro‐mobility shift assay used to draw the plot in C. rIRP1 and rIRP2 indicate assays using the respective recombinant proteins. For each sample, two identical assays were run, one without (−) and one with (+) 2% (v:v) β‐mercaptoethanol (2ME), the latter activating all IRP present in the sample. (C) Active fraction of IRP in KG1a cells under hypoxia. The fraction of active IRP in cell lysates was measured by REMSA as in panel B. The plot represents the ratio without/with 2% 2‐mercaptoethanol for at least three measurements, with SD, since the thiol reveals the latent activity, *that is* that of IRP1 as aconitase or of inactive IRP1/2 by formation of disulfide bridges, in each sample.

In this context, it was relevant to monitor the behavior of the post‐transcriptional regulator of cellular iron homeostasis afforded by IRP1 and IRP2. These cytoplasmic *trans*‐regulators are the RNA‐binding proteins that interact with the non‐coding hairpin structures of several transcripts that are central to iron homeostasis. These hairpin RNA structures, the iron responsive elements (IRE), are present in the 5′ untranslated region of the ferritin subunits and the 3′ one of transferrin receptor 1, for instance. The amounts of these regulatory proteins were measured by western blots after suddenly shifting cells to 1% O_2_ from 20% O_2_ (Fig. 11A). These products were not found to change significantly for *ca*. 30 h. A modest increase of less than 2‐fold could then be detected for IRP1, but this increase cannot be considered statistically significant (One‐way ANOVA *P* = 0.062). The blots analyzed with the anti‐human IRP2 antibody usually showed degradation products (Fig. 11A), including the *ca*. 70 kDa band that was previously characterized [36].

The fraction of IRP that is active in lysates, including KG1a ones, was previously reported to average *ca*. 50% [21]. This fraction can be estimated by reductively activating IRP *in vitro* and comparing the measured activity of the same sample with and without the activating agent, 2‐mercaptoethanol. Upon shifting cells to 1% O_2_, this proportion did not change much over time (Fig. 11B,C) until the end of the incubation period in the same medium (> 60 h) when the carbon source started to become limited (see Fig. 1A). The lack of variation of the IRP activity does explain neither the transient decrease of TFRC mRNA (Fig. 9A), that is stabilized by active IRP, nor the decrease of ferritin‐H that should be constantly repressed by constant IRP activity (Fig. 10). The outcome is thus that the IRP play a minor role in adjusting to abrupt hypoxia in KG1a cells and that other regulatory elements, likely several of them, act on the major participants to iron homeostasis, namely the transferrin receptor and ferritin.

## Discussion

The present work aimed at exploring the behavior of a validated cellular model of minimally maturated myeloid progenitors, the KG1a cell line, after a sudden shift of the oxygen concentration to hypoxic conditions at 1% of O_2_. To some extent, such conditions may mimic the situation occurring when leukemic clones develop in the bone marrow. Indeed, cellular crowding plays a role in the adjustment of the cellular behavior, at least during some phases of cancer development (promotion) because of the competition for substrates [23]. Restricted oxygen supply on a cellular basis, *that is* the oxygen concentration available to each cell, is likely involved. Although these conditions have been studied before with different cells, our data sort out some of the sensitive parameters in KG1a cells and provide a dynamic picture, *that is* time‐based variations, of the cellular adaptation to the newly established conditions.

Yet, it should be kept in mind that low oxygen is considered by some authors as a characteristic property of the environment of hematopoietic cells [26], including stem ones. Care is called to distinguish low O_2_ or physoxia and hypoxia. However, the value of the oxygen partial pressure does not clearly make the difference, and specific markers are required to characterize the actual molecular pattern the studied cells display to adjust to their environment.

Here, hypoxia applied to KG1a cells led to the progressive reduction of the cell fraction engaged in the cell cycle for these strongly multiplying cells under 20% O_2_ and nutrient proficiency. This observation was made before with murine hematopoietic stem cells [37] as well as in human AML cell lines and blasts with consequences on chemosensitivity [38]. However, despite this major response, some molecular features that have been often involved in cancer are insensitive to the drastic lowering of the oxygen tension in KG1a cells. It is the case of the stress markers that have been probed here, including inducible HMOX1. Also, autophagocytosis rather decreased than increased under hypoxia as if cells minimized cell turnover to resist the decrease in proliferation. Active autophagy has been shown to repress initiation in some cancers, including leukemia [39, 40]. Thus, the apparent decrease of the autophagosome markers under hypoxia may be a sign of resistance to cell death for the KG1a model.

Hypoxia has long been known to inhibit mTOR by an AMPK‐independent mechanism: the relevant pathway rather involves REDD1 than activation of AMPK in mouse embryonic fibroblasts [32]. However, other cellular contexts alter this description [41]. In the case of acute myeloid leukemic cells [42], drug‐induced activation of AMPK‐activated mTORC1 and cytotoxicity was correlated with autophagy. These features were not displayed by normal hematopoietic progenitors. However, constitutive activation of mTORC1 in a majority of AML clones has been documented [43]. In the present AML model of KG1a cells, hypoxia did not trigger a clear activation of AMPK (not shown), whereas REDD1 followed HIF1α stabilization (Fig. 5), and mTOR phosphorylation decreased (Fig. 6A). Under such conditions, decreased mTORC1 kinase activity should keep 4E‐BP1 phosphorylated which should in turn repress mRNA translation. Instead, 4E‐BP1 phosphorylation tended to increase, and the total amount of 4E‐BP1 decreased (Fig. 6B,C). These features do not provide a clear picture of the effect on conventional cap‐dependent translation, but the latter may be superseded by hypoxia‐specific translational mechanisms [44].

The metabolic shift of oxidative phosphorylation to glycolysis has long been appreciated under hypoxia, up to becoming a proposed hallmark of cellular behavior in the perturbed environment of cancer cells [45], and it is continuously being refined [46]. However, recent data have rather highlighted the large metabolic flexibility of cancer cells at various stages of development [47, 48]. In the present case of KG1a cells, hypoxia did repress fueling of the mitochondrial TCA cycle by increase of PDK1, and it had some impact on glycolysis as judged by variations of PFKP (Fig. 8A). However, these changes were modest and they did not lead to a major metabolic shift. Indeed, our exploratory oxygraphic experiments showed that permeabilized KG1a cells display a respiratory rate of the order of 1 nmol O_2_ (min)^−1^ (million cells)^−1^, corresponding to *ca*. one‐third of the respiratory capacity measured with the protonophore carbonyl cyanide‐4 (trifluoromethoxy) phenylhydrazone that depolarizes the mitochondrial membrane. Decreasing further the basal respiratory rate under hypoxia is unlikely to have a major influence on cellular energy production since this value is already in the lowest range of O_2_ uptake values measured in a variety of mammalian cells [49].

The partial activity of the IRP was not significantly changed in these cells by the decrease of oxygen availability. The latter observation is apparently discrepant with previous reports. Historically, the IRP1 and IRP2 activities were first found to decrease in a rat hepatoma cell line at 3% O_2_ [50] and to increase in human 293 embryonic kidney and mouse hepatoma cells at 1% O_2_ [51], respectively. However, very soon after, IRP1 activity was rather found to increase at 1% O_2_ in a human hepatoma cell line [52] with seemingly different kinetics than in the former articles. This increase was also observed in the K562 chronic myelogenous leukemia cell line, but with less intensity [52]. The heterogeneity of the response of the IRP to hypoxia was further documented in a later study [53]. Therefore, it appears difficult to draw definitive conclusions as of the expected variations of the IRP activity under hypoxia, and experimental evaluation, as done here with KG1a cells, is needed any time work is carried out in a previously unexplored cellular context.

The IRP are insensitive to the lowering of the oxygen tension available to KG1a cells (Fig. 11), whereas hypoxia is often associated with changes in iron responsive element‐binding activity of IRP1 as recalled above. Accordingly, the major cellular iron importer, the transferrin receptor 1, is stable under the hypoxic conditions applied here, whereas some variations were measured for its mRNA (Fig. 9A). This occurs despite the abundant literature describing the links between hypoxia and iron homeostasis at the systemic and cellular levels with a wealth of molecular actors, and including erythropoiesis, a process involving more committed cell types than the KG1a model. This literature has been recently reviewed [54, 55]. However, drastic collapse of ferritin was measured here (Fig. 10) as previously observed in K562 cells [52]. The disappearance of ferritin is likely not autophagy‐driven [56] since autophagy is not enhanced (Fig. 7A,B). Neither is this lowered concentration mediated by increased IRP activity and subsequently decreased FTH1 mRNA translation since the IRP activity remained stable (Fig. 11B,C). Therefore, the ferritin turnover signs constant or increasing mobilization of iron from this protein to support viability of KG1a cells under hypoxia and lowered stocks with insufficient replenishment. These observations suggest that, although iron homeostasis of KG1a cells fails to thoroughly adjust to massive hypoxia to maintain proliferation for a long time, it depletes internal iron reserves to partly maintain viability. This conclusion, reached here under hypoxia, is in line with the requirement of only minute amounts of iron to sustain viability of cancerous cells in different conditions [20].

**Fig. 12:**
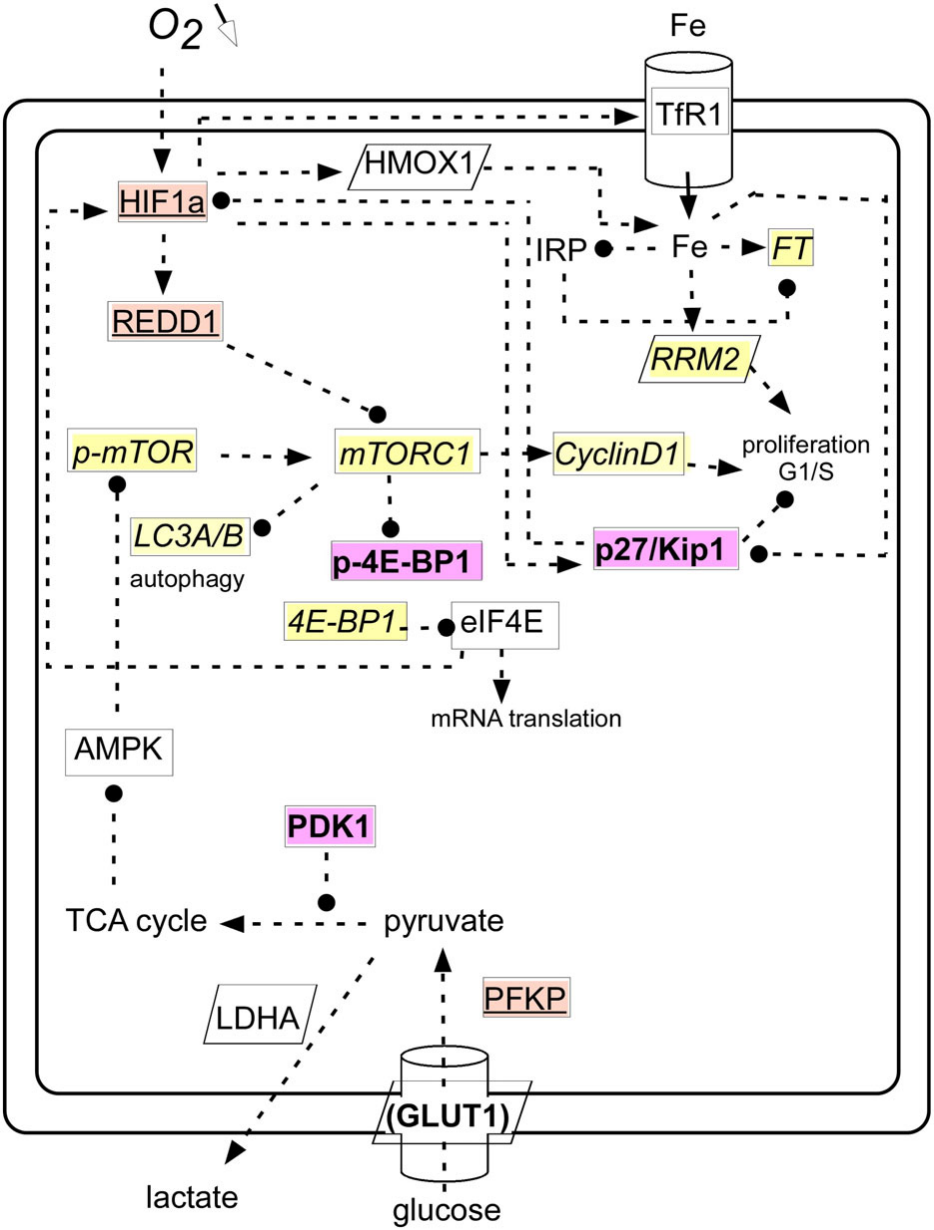
Summary of the observations made under hypoxia for KG1a cells. The Figure lists the measured components with the following code: Plain: no change; italicized and light yellow: Down; bold and pink: Up; underlined and orange: Mixed evolution detailed in the text. The up‐regulation of GLUT1 within brackets indicates that statistical significance was not attained. When the name of the component is boxed, the protein was measured; when the box is parallelepiped, it was the mRNA. The dotted lines indicate direct or indirect interactions among cellular components as found in the literature, at least in some cellular contexts, with an ending arrow meaning positive (activation) interaction, and a spot negative (inhibition) interaction. Close examination of this Figure shows that not all expectations are observed under the present experimental conditions.

The above screening of the molecular consequences of a drastic drop of the available oxygen to the KG1a model is far from comprehensive, particularly regarding the wealth of reactions reported to respond to hypoxia in various cellular types. However, despite this limitation of our work, it appears already that many observations that acquired the status of dogma in the field of hypoxia and redox signaling are not fully confirmed here. This is the case of the supposedly prominent involvement of iron homeostasis [4, 35], for instance. There is no doubt that the KG1a cells did sense the hypoxic stress in the presently reported conditions, since proliferation soon decreased (Fig. 2A) and the fast‐responding transcription factor HIF1α evolved (Fig. 4) as well as REDD1 (Fig. 5). Yet, other expected molecular connections were not observed, even though the shift in oxygen tension from 20 to 1% is major and larger than may happen *in vivo* in most circumstances. The evolution of the measured items and the discrepancies with generally accepted influences are summarized in Fig. 12. Such observations highlight that minimizing the manipulation of cells, as done here by avoiding deliberate gene knock‐out or knock‐in, drug use, or other means to target internal components for instance, lets cells respond in a variety of ways with plenty of molecular strategies to adjust to their new environment.

Only the oxygen concentration as an environmental parameter was changed in the present study. The KG1a cells were able to cope with the challenge for a long time, including a couple of renewals of the growth medium. Extrapolating to the leukemic blasts modeled by the KG1a cells, the diversity of their genetic landscape and the different stages of maturation at which they stall, coupled with their clonal evolution over time [57], are not in favor of a single reliable molecular signature. It would thus be difficult to identify a pre‐defined mechanistic target and the same therapeutic strategy for all patients. Rather, the odds of success against myeloid leukemia are that thorough genomic, transcriptomic, and functional investigations of each patient's blasts or, when readily possible, cancer stem cells are needed to contemplate an actual cure for such a deadly disease as AML. First steps in this direction are being taken [58]. However, even with complete sets of molecular data, the road is still long and full of traps until the aim is reached. Indeed, these data will need to be functionally assembled and understood before any fully informed therapeutic action can be implemented. The presently reported results exemplify, with a very simple system and even with incomplete data, that the task is challenging and stimulating.

## Author contributions

IN‐Z, CL, GES: Investigation, Writing ‐ review and editing; CC‐R: Formal analysis, Investigation, Methodology, Resources, Visualization, Writing ‐ review and editing; J‐MM: Conceptualization, Data curation, Formal analysis, Funding acquisition, Investigation, Methodology, Project administration, Resources, Supervision, Validation, Visualization, Writing ‐ original draft preparation.

## Conflict of interest

The authors declare no conflict of interest.

## Data Availability

The data that supports the findings of this study are available in the article.
